# Sex differences in immune modulation: implications for infection, inflammation, and nutritional supplementation

**DOI:** 10.1186/s13293-026-00857-1

**Published:** 2026-02-18

**Authors:** Marta Araújo, Ana Mendes-Frias, Ricardo Silvestre

**Affiliations:** 1 Life and Health Sciences Research Institute (ICVS), School of Medicine, University of Minho, Braga, Portugal; 2 ICVS/3B’s – PT Government Associate Laboratory, Braga/Guimarães, Portugal

**Keywords:** Immunology, Inflammatory diseases, Infectious diseases, Immunonutrition, Sex-based differences

## Abstract

The immune system is central to maintaining homeostasis and orchestrating defense against infection and the regulation of inflammatory responses, yet its activity is far from uniform across individuals. Sex differences profoundly shape immune responses, with sex hormones driving distinct patterns of susceptibility to infectious diseases and inflammatory conditions. Nutrition further adds a powerful layer of modulation: vitamins, amino acids, and other bioactive compounds influence immune function and disease outcomes, often in a sex-dependent manner. The microbiome, whose composition is itself influenced by sex, is a critical regulator of both intestinal and systemic immune disorders, making it an attractive target for therapeutic intervention. In this review, we examine the dynamic interplay between sex, nutrition, and the immune system, emphasizing their combined impact on infection, inflammation, and immunomodulation. A deeper understanding of these interactions will be key to advancing personalized nutritional and therapeutic strategies designed to optimize immune health.

## Introduction

Biological sex is a fundamental determinant of immune system function, influencing susceptibility to infection, inflammatory regulation, and the development of autoimmunity. Sex-based differences arise from a complex interplay of genetic factors, sex hormones, and environmental influences, and they shape both innate and adaptive immune responses across the lifespan [1]. These immunological differences can be protective in some contexts while predisposing individuals to pathology in others, underscoring their relevance for disease outcomes and therapeutic responses.

Sex-dependent immune variation has important implications across multiple domains, including pathogen defense, inflammatory control, autoimmunity, and responses to nutritional and microbiome-based interventions. For example, males and females often differ in susceptibility, disease severity, and treatment outcomes in infectious diseases, inflammatory disorders, and autoimmune conditions [2–4]. Recently, COVID-19 has served as a clear example of how sex matters, with men showing higher morbidity and mortality compared to women – differences directly linked to immune function [2, 5]. In this review, in addition to COVID-19, we will discuss other infections caused by parasites (*Leishmania* spp.), bacteria (*Mycobacterium tuberculosis* and *Streptococcus* spp.) and fungi (*Cryptococcus* spp.). Beyond infection, the influence of sex has been extensively investigated in the context of autoimmune diseases and chronic inflammatory disorders, where heightened immune activation can be both beneficial for pathogen control and detrimental through increased inflammatory or autoimmune pathology [6, 7]. In general, women generally have more a activated immune system, which enhances pathogen clearance but also contributes to a higher risk of autoimmunity. Consequently, autoimmune diseases such as systemic lupus erythematosus (SLE), rheumatoid arthritis (RA), and multiple sclerosis (MS) occur more frequently in women than in men [8–10]. Sex differences have also been widely explored in inflammatory conditions, particularly in inflammatory bowel diseases (IBD) [11]. In ulcerative colitis (UC), disease management is especially challenging in women as fluctuations in hormonal levels strongly affect both symptomatology and remission patterns [12, 13]. Building on this evidence, our group has investigated the role of sex hormones in UC, with focus on their interplay with supplementation regimens [14]. In this review, we will briefly discuss the mechanisms underlying sex-based differences in autoimmune diseases, examine the contribution of sex hormones and nutritional supplementation in UC, and highlight the critical role of the microbiome in these conditions.

In recent years, dietary and microbiome-targeted interventions have gained increasing attention as modulators of the immune function [15–17]. Beyond the use of chemical supplements such as vitamins, amino acids, and fatty acids, specific dietary regimens have been designed as anti-inflammatory strategies to address both IBD and systemic inflammatory and infectious conditions [15, 18, 19]. More recently, microbiome-targeted approaches have emerged, with prebiotics, probiotics, and postbiotics being employed as adjuvants to conventional therapies, marking a new era in immune modulation [20, 21]. Importantly, the efficacy of these interventions is increasingly recognized to be sex dependent.

In this review, we first outline the biological mechanisms underlying sex-based differences in immune responses. We then examine how these differences shape susceptibility to infection, inflammation, and autoimmunity, with particular attention to the roles of nutrition, supplementation, and microbiome. Finally, we discuss the clinical implications of these interactions and their relevance for developing personalized, sex-aware therapeutic strategies.

## Sex-based differences in immune function, infection susceptibility, and inflammatory responses

### Sex- biased differences in immune responses

**Fig. 1:**
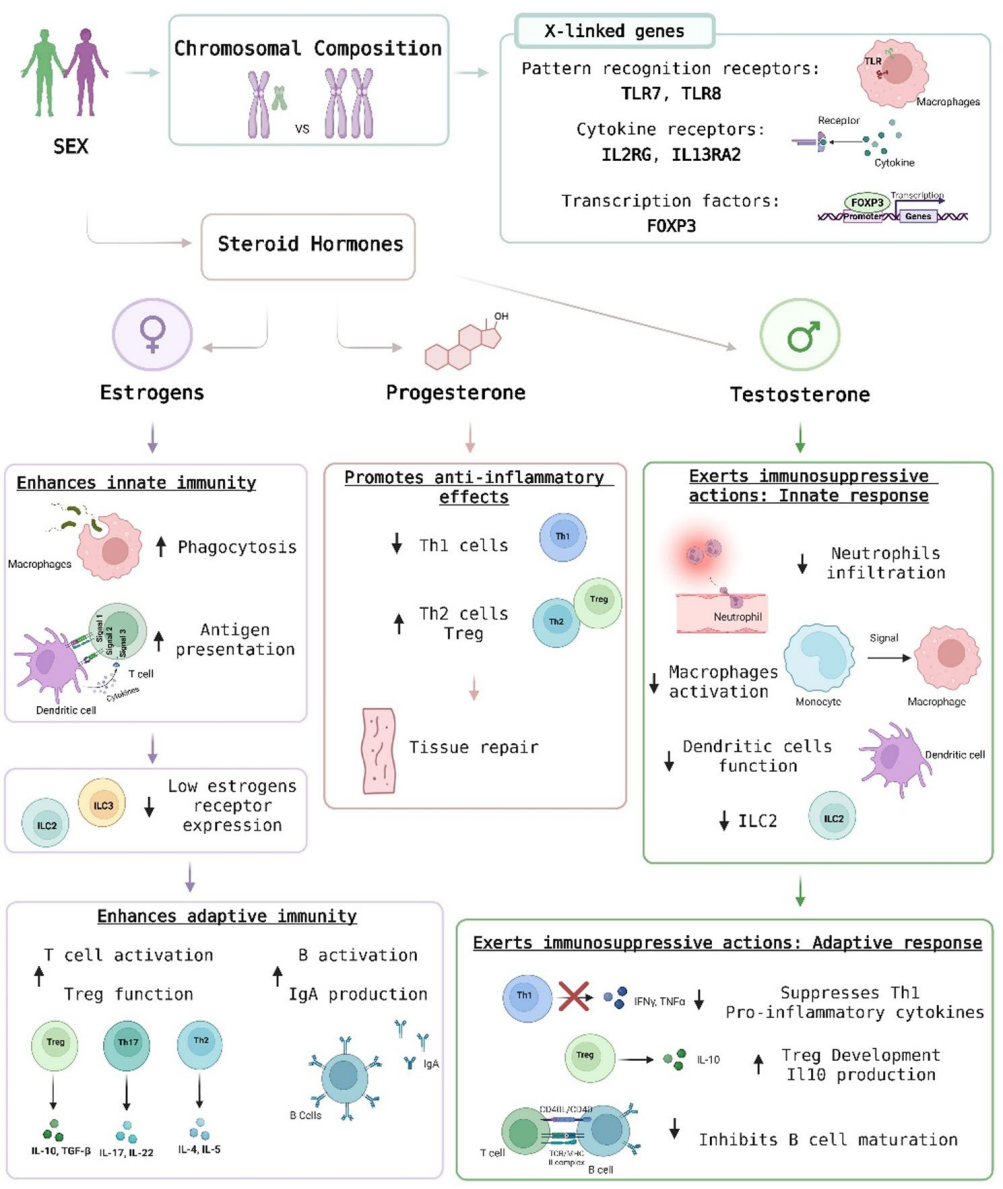
Mechanisms of sex-based immune modulation through chromosomal and hormonal pathways. Biological sex influences immune responses via both genetic and hormonal mechanisms, shaping innate and adaptive immunity. Chromosomal composition (XX in females, XY in males) leads to differential expression of immune-related genes located on the X chromosome, such as pattern recognition receptors (TLR7, TLR8), cytokine receptors (IL2RG, IL13RA2), and the transcription factor FOXP3. These genes can escape full X inactivation in females, resulting in heightened immune responsiveness compared to males. In addition, sex steroid hormones—estrogens, progesterone, and testosterone—modulate immune cell activation and function. Estrogens enhance innate immunity by promoting phagocytosis in macrophages, increasing antigen presentation by dendritic cells, and sustaining activity despite low receptor expression on innate lymphoid cells (ILC2 and ILC3). Estrogens also enhance adaptive immunity by boosting T cell activation and skewing differentiation toward Th17 and Th2 phenotypes, while increasing Treg function, B cell activation, and IgA production. Progesterone exerts primarily anti-inflammatory effects, suppressing Th1 differentiation and promoting Th2 and Treg responses. It also facilitates tissue repair mechanisms. Testosterone exerts predominantly immunosuppressive effects. In the innate immune system, testosterone reduces neutrophil infiltration, inhibits macrophage and dendritic cell activation, and suppresses ILC2 responses. In adaptive immunity, testosterone inhibits Th1 differentiation and production of pro-inflammatory cytokines (IFNγ, TNF-α), while promoting Treg development, increasing IL-10 production, and reducing B cell maturation

Biological sex exerts a profound influence on immune regulation by shaping the balance between pro-inflammatory and anti-inflammatory pathways. “Sex” refers to the biological distinctions between males and females, including chromosomal composition, reproductive anatomy, and levels of sex steroid hormones, which drive physiological and immunological differences [22] (Fig. 1). Testosterone is the predominant sex hormone in males, while estrogen and progesterone are the primary sex hormones in females. Both sexes produce all three hormones at different concentrations, and local tissue conversion (e.g., aromatase activity) further shapes immune exposures [23]. These hormones play significant roles in shaping both innate and adaptive immunity [1, 24]. Their immunomodulatory effects occur via interactions with hormone receptors on immune cells and via additional systemic effects on signaling pathways that control cell activation, differentiation, and cytokine production as well as hematopoiesis, barrier integrity, metabolism, and the microbiota [25, 26].

At the genetic level, females carry two X chromosomes, while males have one X and one Y chromosome [27]. To prevent overexpression of X-linked gene transcription, one X chromosome is randomly inactivated in each female cell [27]. The X chromosome is enriched in immune-related genes, and variations in their expression can lead to sex-specific immune responses. Dysregulation of X-linked genes contributes to autoimmunity, chronic inflammation, and altered lymphocyte function [28, 29]. For example, X-linked genes contribute to higher expression of immune-related molecules in females, including pattern recognition receptors such as Toll like receptor (TLR) 7 and TLR8, cytokine receptors like interleukin-2 receptor gamma chain (IL2RG) and interleukin-13 receptor alpha 2 (IL13RA2), and transcription factors such as forkhead box protein P3 (FOXP3), all of which are critical for pathogen recognition and downstream immune modulation [30–32].

Innate immunity is the body’s first line of defense, providing a rapid, non-specific response to invading pathogens by recognizing common molecular patterns shared by microbes. Its main cells—such as neutrophils, macrophages, dendritic cells, natural killer (NK) cells, and innate lymphoid cells (ILCs)—function to engulf and destroy pathogens, trigger inflammation, and activate the adaptive immune response [33]. ILCs contribute by rapidly producing cytokines that support early inflammatory responses, tissue repair, and barrier defense [34]. Sex hormones have cell-type-specific effects on innate immunity. At physiologic levels, testosterone generally exerts immunoregulatory/anti-inflammatory effects (particularly in the gut mucosa) [35]. It reduces neutrophil infiltration, inhibits macrophage activation, and dampens dendritic cell (DC) function by lowering major histocompatibility complex class II (MHCII) and CD86 expression and suppressing TLR-induced cytokine production [36, 37]. Testosterone also suppresses type 2 ILCs responses, which are essential for anti-helminth immunity, allergy and tissue repair [38, 39]. Estrogen strongly modulates innate immune cells, shaping both their inflammatory and regulatory functions. In DCs, it promotes differentiation, survival, and maturation, enhances MHC class II and co-stimulatory molecule expression, and increases cytokine production (IL-6, IL-12, type I interferons), boosting antigen presentation, T-cell activation, and Th1/Th17 responses [40, 41]. In neutrophils, estrogen enhances recruitment, survival, chemotaxis, phagocytosis, and reactive oxygen species production, strengthening pathogen killing while also modulating inflammation [42, 43]. Regarding macrophages, estrogen influences activation, polarization, and cytokine secretion, promoting antimicrobial and inflammatory responses (tumor necrosis factor alfa (TNF-α), IL-1β, IL-6) while also supporting tissue-repair or anti-inflammatory (M2-like) phenotypes depending on context [44, 45]. ILCs are similarly affected, with estrogen enhancing ILC1 and ILC3 cytokine production (IFN-γ, IL-17, IL-22) for early inflammation and mucosal defense, while modulating ILC2 activity to support type 2 immunity and tissue repair [46, 47]. For more detail on the direct impact of estrogen the different cells types check these reviews [24, 40, 44, 48, 49]. Overall, estrogen strengthens innate immune activation and pathogen defense but can also contribute to heightened immune reactivity and increased susceptibility to inflammatory or autoimmune conditions in females.

Adaptive immunity is a specific defense system that develops after antigen exposure and generates immunological memory, enabling faster and stronger responses upon re-infection. It is mediated mainly by T and B lymphocytes: Th1 and Th17 cells promote inflammation, Treg cells exert anti-inflammatory and tolerance-maintaining functions, CD8⁺ T cells kill infected cells, while B cells produce antigen-specific antibodies [50, 51]. The adaptive immune system is similarly affected by sex hormones. Testosterone suppresses T helper 1 (Th1) differentiation and pro-inflammatory cytokines, such as interferon gamma (IFN-γ) and TNF-α, and inhibits IL-17 production by Th17 cells [52, 53], while promoting regulatory T cell development and IL-10 production [54]. In B cells, testosterone inhibits maturation and IgA-producing plasma cells in intestinal mucosa [55]. On the other hand, estrogen enhances T-cell activation and can bias toward Th17/Th2 differentiation, increasing IL-17 and IL-4/IL-5, and it boosts chemokine-receptor expression that facilitates T-cell homing to mucosal tissues [56, 57]. Additionally, estrogen enhances the function of Tregs, potentially promoting immune tolerance [58], and augments B-cell activation, proliferation, survival, and antibody (including IgA) production [55]. Progesterone suppresses Th1 responses and promotes Th2 and Treg differentiation, contributing to immune tolerance and tissue repair [59, 60]. It also modulates antibody isotype switching and reduces B cell activation [55]. Overall, framing hormone effects along pro-inflammatory (Th1/type-1) versus anti-inflammatory/regulatory (Th2/Treg) axes clarifies how biological sex shapes adaptive immunity in a context dependent manner.

### Sex-biased differences in host response to infectious diseases

In females, immune responses are further shaped by cyclical hormonal fluctuations across the menstrual cycle, which dynamically influence inflammatory tone and pathogen control [61]. During the luteal phase (~ 14 days), rising progesterone shifts immunity toward Th2/regulatory profiles, transiently blunting Th1 cytotoxicity and creating a day window in which cellular pathogen clearance may be reduced while barrier repair is enhanced [62–64]. Importantly, these hormonal oscillations illustrate the broad, context-independent impact of sex steroids on immune regulation in both sexes. In contrast, sex biased differences in infection outcomes arise when these generalized hormonal effects intersect with pathogen specific requirements for protective immunity (for example, strong Th1 versus type 2 or regulatory responses). Human epidemiologic studies and animal models suggest that, during such periods of increased susceptibility, females may be more vulnerable to infections that require strong Th1-mediated immune clearance, including viral infections such as influenza A virus, as well as bacterial pathogens like *Mycobacterium tuberculosis* [65, 66]. More broadly, sex-based immune bias manifests most clearly in inflammatory processes and infection outcomes. Females typically exhibit more robust humoral and cellular adaptive responses than males, resulting in faster pathogen clearance in both human and animal studies [67–69]. However, this heightened immunity also predisposes females to excessive inflammation, cytokine storms, and autoimmune disease [70]. Sex hormones are the principal modulators of these differences, shifting immunity along pro-inflammatory (e.g., Th1/type 1) versus anti-inflammatory/regulatory (Th2/Treg) axes [71]. Viral infections provide a clear example of how general hormonal modulation translates into sex-specific patterns of susceptibility and outcome. Estradiol enhances early antiviral immunity by positively regulating Toll-like receptor–mediated responses in plasmacytoid dendritic cells through estrogen receptor-α signaling, leading to increased type I interferon production in vivo [72–74]. In females, this estradiol driven boost in type I IFN strengthens early viral control, however if sustained or excessive, it may amplify downstream inflammatory cascades and contribute to immunopathology. Thus, the same hormone-mediated mechanism that promotes efficient pathogen sensing and clearance can, in a sex-specific context, also increase the risk of immune-mediated tissue damage.

Sex-based differences are well documented across viral infections (e.g., influenza, COVID-19, hepatitis B) [51, 75]. Focusing on COVID-19, females generally mount stronger T-cell responses, whereas males show higher innate pro-inflammatory cytokines (e.g., IL-8, IL-18, CXCL10) and, on average, weaker T-cell responses [2, 76]. Together, these observations indicate that males may exhibit impaired early antiviral type I IFN signaling and suboptimal T-cell control, while simultaneously mounting heightened inflammatory cytokine/chemokine responses that drive pathology. These findings suggest sex-linked variation in both the magnitude and the quality of antiviral immunity during SARS-CoV-2 infection [2]. Ex vivo stimulation of peripheral blood mononuclear cells (PBMCs) from healthy human donors with SARS-CoV-2 shows greater early activation of dendritic and monocyte subsets in females at ~ 24 h, consistent with a stronger early antiviral response [5]. In contrast, males showed a delayed type I IFN response at later time points (with elevated IFN-α appearing around ~ 1 week), along with increased CXCL-10 secretion—a chemokine linked to lung inflammation and disease severity [5]. These sex-stratified patterns vary by age, comorbidities, and treatment era, which contributes to heterogeneity across studies [2]. Evidence from gender-affirming hormone therapy further supports a direct role for sex hormones in shaping antiviral immune programs relevant to SARS-CoV-2 responses. In humans receiving testosterone therapy, type I IFN responses in pDCs/monocytes are attenuated, while natural killer cells show increased TNF-α, IL-6, IL-15 as well as IFN-γ, highlighting the immunomodulatory effects of testosterone relevant to antiviral responses, including COVID-19 [36, 77].

Beyond acute infection, sex-specific mechanisms also appear to influence susceptibility to post-viral syndromes such as Long COVID (post-acute sequelae of SARS-CoV-2 infection). A recent multi-omic study in humans [78] identified distinct sex-specific immune mechanisms underpinning long-term post-COVID manifestations. Males who develop long COVID displayed higher activation of transforming growth factor beta (TGF-β) pathway during the acute phase, a pathway known to suppress antiviral T-cell responses and promote tissue remodeling and fibrosis, thereby potentially impairing viral clearance and contributing to prolonged immune dysfunction [78]. In contrast, females with persistent symptoms showed reduced TGFB1 levels and increased expression of X-inactive specific transcript (XIST), a long non-coding RNA involved in X-chromosome inactivation and implicated in autoimmune pathogenesis via dysregulation of X-linked immune genes, suggesting that altered X-linked immune regulation may contribute to sustained immune activation and autoimmune-like features in females with long COVID [78]. Together, these findings indicate that males and females may develop long COVID through distinct, sex-specific immunopathologic pathways.

Across both sexes, sustained monocyte activation and increased nuclear factor κB (NF-κB) signaling are observed throughout acute and convalescent phases, indicating prolonged innate immune activation [78]. In individuals of both sexes with persistent symptoms, reduced expression of ETS1, a transcription factor that restrains excessive T-cell activation and promotes immune tolerance, together with elevated IL-4 expression in T cells, is indicative of a dysregulated, Th2-skewed immune profile. These findings align with epidemiologic evidence indicating that females, particularly those aged 40–54, are 1.3–1.5 times more likely than males to experience Long COVID, with the interplay of hormonal and X-linked genetic factors likely contributing to this female-predominant risk [78]. Estimates vary by cohort definitions, vaccination status, and variant period.

Similar sex-dependent immune patterns are observed in parasitic infections. In Brazil, where visceral leishmaniasis (VL) remains a growing public health challenge, clinical and experimental data indicate male-biased susceptibility linked to sex hormones [79]. In humans, epidemiological surveillance consistently reports higher incidence, more severe disease, and worse outcomes in males, particularly during and after puberty, even beyond behavioral exposure [79, 80]. Males with VL tend to present higher parasite loads, larger spleen and liver volumes, more pronounced anemia, and greater clinical severity compared to females of the same age [79, 80]. Male hamsters infected with distinct *Leishmania* species developed significantly larger and more severe lesions than females, associated with increased expression of IL-4, IL-10, and TGF-β—cytokines that facilitate disease progression [81, 82]. Similarly, androgens like dihydrotestosterone (DHT) can suppress protective immunity in mice, promoting *L. mexicana* proliferation and impairing macrophage microbicidal activity [83]. Conversely, in females, 17β-estradiol improves resistance by enhancing nitric oxide production in macrophages [84], while IL-12p70 plays a critical role in driving protective immunity in female macrophages [85]. Progesterone shifts toward Th2/Treg programs, suppressing IFN-γ and TNF-α and stabilizing mast cells; which may limit immunopathology yet, in intracellular infections, reduce pathogen clearance during the luteal phase [64, 86]. Notably, sex differences persist even after adjusting for nutritional status [79, 80]. Together, these data support androgen-linked susceptibility and estradiol-associated protection in leishmaniasis, with progesterone exerting context-dependent immunomodulation [79, 80]. These hormone-dependent effects converge on macrophage effector functions, cytokine balance, and T-cell polarization, collectively shaping sex-biased outcomes in leishmanial infection.

Comparable mechanisms extend to bacterial and fungal infections. In a mouse model of *Streptococcus agalactiae* pneumonia, males show higher pro-inflammatory cytokines and an elevated M1/M2 macrophage ratio, linked to lower X-linked miR-223-3p [87]. In human *Cryptococcus neoformans* infection, male PBMCs show reduced CD3⁺, CD4⁺, and CD8⁺ T cell and permit greater fungal proliferation, pointing to an inherent T cell deficit in males that may explain their higher cryptococcosis incidence [88, 89]. Estrogens enhance innate and adaptive antimycobacterial defenses by promoting macrophage activation, increasing nitric oxide production, and upregulating Th1/pro-inflammatory cytokines such as IFN-γ and TNF-α, thereby favoring the Th1-dominant response crucial for controlling *Mycobacterium tuberculosis* [90]. In murine models, ovariectomy increases lung bacterial burden and pathology, whereas estradiol replacement restores control and raises inducible nitric oxide synthase (iNOS) and IFN-γ⁺ CD4⁺ T cells [91]. In contrast, testosterone suppresses macrophage activation and skews toward anti-inflammatory M2 polarization, reducing production of nitric oxide and Th1 cytokines; male mice show lower IFN-γ/TNF-α and worse lung pathology compared to females, consistent with reduced bacterial clearance [92]. Overall, sex hormones orchestrate immune responses across a spectrum of infections, driving distinct trajectories of susceptibility, disease severity, and recovery in males and females. Embracing these biological insights holds the promise of more personalized and effective interventions for infectious diseases.

Sex-based differences also extend into the clinical sphere, influencing responses to immunotherapies, vaccine efficacy, and drug side effects. Females often develop stronger responses to vaccines but also experience more frequent side effects, whereas males may require higher doses to reach equivalent levels of protection [93]. Similarly, estrogens typically enhance immune defense, while androgens suppress key immune function, potentially increasing disease severity in males [52]. Overall, males generally exhibit relatively blunted antiviral and antibacterial defenses, which makes them more susceptible to severe and chronic infections [94]. Whether this immune dampening confers protection against immunopathology or represents a disadvantage in pathogen clearance remains incompletely understood and warrants further investigation. Hormone-linked differences across major pathogens are summarized in Table 1.

**Table 1:** Effects of sex hormones on immunity against selected pathogens

Pathogen	Estrogens	Progesterone	Testosterone	References
**Influenza A virus**	• Enhances Th1-mediated viral clearance; • Upregulates TLR7, type I IFNs; • Improves antiviral T cell responses.	• Skews toward Th2/Treg; • Creating a period of vulnerability with reduced Th1 cytotoxicity.	• Suppresses antiviral T and B cell responses; • Increases disease severity.	[51, 75]
**SARS-CoV-2 (COVID-19)**	• Promotes TLR7 expression; • Enhances type I IFNs and effective T cell responses; • Protects against hyperinflammation.	• Skews toward Th2/Treg; • Tissue repair mechanisms; Reduced Th1-driven viral clearance.	• Dampens antibody and T cell responses; • Increases pro-inflammatory cytokines (IL-8, IL-18).	[2, 5, 36, 72, 76]
**Long COVID (Post-viral syndrome)**	• Associated with reduced TGF-β1; • Increased XIST expression (autoimmune-like signatures); • Th2 skew.	Not specified.	• Elevated TGF-β pathway activation, sustained monocyte NF-κB signaling; • Th2 dysregulation.	[78]
***Leishmania spp.*** **(Visceral leishmaniasis)**	• Enhances nitric oxide production in macrophages; • Increases IL-12p70; • Promotes Th1 protective immunity.	• Skews toward Th2/Treg; • Suppresses Th1 cytokines (IFN-γ, TNF-α); • Promotes tolerance.	• Increases IL-4, IL-10, TGF-β; • Suppresses macrophage microbicidal activity, worsens disease severity	[64], 79– [81, 83, 85, 86]
***Mycobacterium tuberculosis***	• Enhances macrophage activation; • Nitric oxide production; • IFN-γ and TNF-α; • Promotes Th1 immunity.	• Skews toward Th2/Treg; • Reduces Th1-driven antimycobacterial response	• Suppresses macrophage activation; • Skews M2 phenotype; • Lowers IFN-γ and TNF-α; • Impairs bacterial clearance.	[89–92]
***Cryptococcus neoformans***	• Higher T cell frequencies (CD3+, CD4+, CD8+); • Reduced fungal proliferation.	Not specified	• Reduced T cell frequencies; • Impaired fungal clearance. • Higher incidence in males.	[88]
***Streptococcus agalactiae*** **(pneumonia)**	• Higher miR-223-3p levels in females limit inflammatory macrophage polarization.	Not specified	• Lower miR-223-3p in males leads to excessive pro-inflammatory macrophages, worse lung inflammation.	[87]

### Sex bias across autoimmune diseases

As mentioned before, females exhibit stronger antibody responses, which contributes to the increased prevalence of autoimmune diseases compared to male. SLE is a classic example, with a female-to-male ratio of approximately 9:1 [95]. SLE is a chronic autoimmune disease affecting multiple organs, with symptoms that vary widely and often come in flares, including fatigue, fever, joint pain, skin rashes (especially malar rash), oral ulcers, hair loss, kidney problems, blood abnormalities, and sometimes heart, lung, or nervous system involvement [96]. At a molecular level, overexpression of X-linked genes due to incomplete inactivation of the second X chromosome is one of the mechanisms responsible for sex-specific differences in SLE [10]. Epigenetic studies in T and B cells from SLE patients revealed sex- and cell-type-specific epigenetic modifications, although their effects on IFN-driven immune responses remain to be fully elucidated [10, 97]. In females, elevated levels of sex hormones are associated with increased production of type I and type II IFN and mitochondrial dysfunction, including changes in adenosine triphosphate (ATP) production, reactive oxygen species (ROS) release and disturbed calcium homeostasis, mechanistic features that contribute to SLE pathogenesis [10]. Despite the lower prevalence of SLE in males, disease severity tends to be greater, with males more frequently developing lupus nephritis, the most severe clinical form of SLE. This manifestation is characterized by immune complex-mediated glomerular inflammation, and it can evolve to end-stage renal disease (ESRS), a life-threatening complication [98].

Another autoimmune disease with clear sexual dimorphism is rheumatoid arthritis (RA). RA is more common in females with female-to-male ratio of approximately 3:1 [8, 99]. This sex imbalance has limited the design of sex-based studies, as female predominance introduces a confounding variable [99]. RA is a chronic, systemic inflammatory joint disease characterized by the presence of rheumatoid factor (RF) and anti-citrullinated proteins antibodies (ACPAs) [99], which drive joint destruction and systemic inflammation. Clinically, RA presents symmetrical polyarthritis with extra-articular manifestations, progressing to joint damage and irreversible disability [99]. Females present worst symptomatology and disease activity, unleashing a poor outcome [8]. In contrast, males are more likely to achieve remission and show longer survival times [8]. Estrogen has different immunomodulatory effects in RA. Indeed, hormonal fluctuations are described to have an impact in disease activity and progression, for instance during pregnancy women have reduced disease activity due to the high levels of estrogens, while after menopause they have increased risk to develop the disease [100]. Mechanistically, estradiol reduces Th17 cell accumulation in joints while increasing their retention in lymph nodes, lowering IL-17–mediated synovial inflammation via ERα signaling [100]. Also, estrogen enhances regulatory T cells, promoting anti-inflammatory responses, and modulates cytokine levels by decreasing pro-inflammatory cytokines (TNF‑α, IL‑1β, IL-6) and increasing anti-inflammatory mediators (IL-10) [101]. In RA, estrogen modulates B cells by increasing IgG Fc sialylation and promoting a less inflammatory antibody profile, while the decline in estrogen after menopause reduces this protective modification and contributes to more pathogenic autoantibody activity [102]. These combined effects help explain why RA often improves during pregnancy, when estrogen is high, and flares postpartum when estrogen drops, highlighting the role of hormonal regulation in disease activity.

Multiple sclerosis (MS) is a chronic, immune mediated demyelinating disease of the central nervous system characterized by neuroinflammation and neurodegeneration [103]. Both autoreactive T and B cells are the important players in this disease [103]. Strong sex-based differences have been reported in its prevalence, clinical course and response to treatment [9]. Women are disproportionately affected, with female-to-male ratio of approximately 3:1, and this disparity has been increasing over recent decades [9]. Beyond prevalence, sex also influences disease progression: while women more frequently develop the relapsing-remitting form of MS, men tend to experience less inflammatory activity but fast disability, more severe brain atrophy, and higher risk of entering the progressive phase earlier [104]. Women often show stronger immunological response to disease-modifying therapies, yet treatment strategies must account for reproductive considerations, pregnancy, and hormonal fluctuations, which can significantly impact the disease activity [105]. Hormonal and genetic factors are thought to contribute to this imbalance, with estrogen and other sex hormones modulating T cell polarization and neuroprotection [106]. Specifically, estrogens - particularly at pregnancy or therapeutic levels - shift the immune response away from pro-inflammatory Th1 and Th17 cells towards anti-inflammatory Th2 cells and regulatory T cells, with associated increase in IL-10 and reduction in cytokines such as TNF-α and IFN-γ; these changes correlate with fewer relapses and reduced MRI inflammatory activity [107]. Progesterone directly suppresses immune activation in MS by inhibiting NF-κB signaling in immune cells, reducing production of pro-inflammatory cytokines [108]. Progesterone promotes regulatory T-cell development and decreases IFN-γ and IL-17, limiting autoreactive T-cell responses central to MS pathology [109]. In men with MS, testosterone directly suppresses pathogenic immune activity. Testosterone treatment reduces CD4⁺ T-cell numbers and lowers pro-inflammatory cytokines such as IL-2, while increasing the immunoregulatory cytokine TGF-β1 [110]. Overall, sex represents a key biological variable in MS, shaping susceptibility, clinical expression, and therapeutic outcomes, and should therefore be considered in the development of personalized management strategies.

Sex dimorphism is widely observed in the pathogenesis of ulcerative colitis (UC). While incidence rates vary between populations, the observed sex differences are likely influenced by cultural, geographic, and environmental factors [111]. Clinically, sex influences disease progression, treatment response and extraintestinal complications. Females usually experience longer disease duration and lower remission rates compared to males [112]. Regarding treatment, females more often require corticosteroid therapy [113] and are at greater risk of azathioprine intolerance, hypersensitivity reactions, and reduced response to biologics like adalimumab [114, 115]. Additionally, disease activity and response to therapy in women are strongly influenced by hormonal status [12, 116]. During the fertile period, fluctuations in estrogen and progesterone across the menstrual cycle can exacerbate gastrointestinal symptoms and, in some cases, affect response to treatment [117–119]. Pregnancy introduces an additional complexity: while some women experience remission, others show increased relapse risk, particularly in the postpartum period, likely due to abrupt hormonal shifts [120–122]. Menopause also represents a critical stage, as declining estrogen levels have been associated with changes in immune regulation and gut barrier function, potentially altering both disease course and therapeutic outcomes [123, 124]. Overall, hormonal transitions in women modulate UC activity, relapse risk and treatment responses, underscoring the importance of personalized strategies across reproductive stages. Extraintestinal manifestations are also influenced by sex dimorphism. Females more commonly develop arthritis, erythema nodosum and ocular manifestations [113], while males are at higher risk of primary sclerosing cholangitis and ankylosing spondylitis [125]. Notably, females with UC have higher mortality due to cardiovascular and pulmonary complications [113].

## Nutritional supplementation as a modulator of immune function and inflammation: sex-specific considerations

Environmental factors, including diet, drug consumption, nutrition, and microbiome composition can influence immune responses and modulate gene expression, including silenced genes on the inactive X chromosome [97, 126]. Nutritional supplementation has been shown to support immune defenses, mitigate chronic inflammation, and enhance resistance to infection. Importantly, emerging evidence suggests that these immunological effects of nutrients are shaped by biological sex, reflecting sex-specific differences in immune responsiveness, hormone regulation, and nutrient metabolism.

### Feeding immunity: can nutrition mitigate sex differences in infection?

Beyond general health, targeted nutritional supplementation has been shown to strengthen immune responses in vulnerable or nutrient-deficient individuals (summary in Table 2). Nutritional supplementation represents a targeted, evidence‑informed strategy to enhance host immune competence and attenuate the risk and severity of infectious diseases, particularly in contexts where habitual dietary intake is inadequate. Given that specific micronutrients and bioactive lipids exert immunomodulatory effects through diverse cellular and molecular pathways, including cytokine regulation, antioxidant defense, and resolution of inflammation, the potential for supplementation to beneficially modulate infection outcomes is well‑supported by mechanistic and clinical evidence [127]. Despite well-established sex-based immune differences, most nutritional intervention studies in infection fail to include sex-stratified analyses or dosing considerations. This represents a critical gap in the current evidence base and highlights the need for future research to elucidate whether tailored supplementation regimens, aligned with sex‑specific immune phenotypes, can optimize infection prevention and management.

#### Nicotinamide

Nicotinamide, a form of vitamin B₃, demonstrated potent antiparasitic activity in a *L. infantum* murine model, reducing liver and spleen burdens by 79.7% and 86.7%, respectively, and improving organ pathology [128]. Mechanistically, nicotinamide reduced pro-inflammatory cytokines and enhanced lipid metabolism, suggesting it can both suppress parasitic proliferation and restore metabolic balance [128]. This dual action highlights its potential as a safe and cost-effective adjunct therapy for VL. However, since the study was conducted only in females, it raises important questions about how nicotinamide supplementation interacts with immune differences between sexes. Higher susceptibility to VL in males, driven in part by androgen-mediated immune modulation and a weaker Th1 response [128], underscores the need to evaluated whether nicotinamide’s immunomodulatory and metabolic effects can mitigate this disadvantage. Assessing supplementation strategies through a sex-informed lens is therefore essential to ensure that potential benefits are appropriately applied to both sexes.

#### Vitamin C

Vitamin C is a key micronutrient for maintaining immune competence, particularly during infections. It enhances epithelial barrier integrity, promotes chemotaxis and microbial killing by neutrophils, supports lymphocyte proliferation, and regulates pro- and anti-inflammatory cytokines [129]. Supplementation of 200 mg per day or more can shorten the duration and reduce the severity of respiratory infections, especially in physically active individuals [18, 129]. Clinical and pre-clinical data show that infections deplete vitamin C levels rapidly due to increased metabolic demand and oxidative stress, making adequate intake crucial in both prevention and treatment settings [130]. Evidence reviewed from randomized controlled trials and observational studies indicates that vitamin C supplementation can reduce the risk and severity of respiratory infections, including pneumonia and COVID-19, shorten hospital stays, improve oxygenation, and lower inflammatory markers such as C-reactive protein and IL-6 in critically ill patients. However, most clinical studies do not report sex-stratified outcomes, limiting conclusions regarding sex-specific efficacy. Available data suggest that baseline vitamin C status and immune responses may differ between males and females, but these differences are rarely analyzed explicitly in clinical trial designs. Vitamin C has also shown promise as an adjunctive therapy in tuberculosis, demonstrating host-directed immunomodulatory effects and antimycobacterial activity in experimental models [130–133]. In vitro and animal studies reveal that high-dose vitamin C can exert antimycobacterial activity, including against drug-resistant *M. tuberculosis*, largely through the induction of oxidative damage, while enhancing macrophage-mediated immunity. However, these antimycobacterial effects have primarily been observed under experimental conditions and do not support the use of vitamin C as a standalone sterilizing agent in clinical tuberculosis treatment. Instead, available evidence suggests that vitamin C may act synergistically with standard anti-tuberculosis drugs and contribute to host-directed immunomodulation, potentially improving treatment efficacy and immune control of infection [130–133]. TB patients often exhibit lower vitamin C levels, but whether sex-related differences in vitamin C status or immune responses influence TB outcomes remains unclear, highlighting an important gap for future investigation [130–133].

**Table 2:** Effects of nutritional supplements on immune function and infection outcomes

Supplement	Immune cell Impact & mechanism	Infection outcome	Sex-specific notes	References
**Nicotinamide (Vitamin B₃)**	↓ Pro-inflammatory cytokines, enhances lipid metabolism, restores immune–metabolic balance	↓ *Leishmania infantum *burden (liver: -79.7%, spleen: -86.7%), improved pathology in VL murine model	Tested only in females; impact in males with weaker Th1 response remains unknown	[128]
**Vitamin C**	↑ Neutrophil chemotaxis, killing, lymphocyte proliferation, epithelial barrier; regulates cytokines (↓ IL-6, CRP)	↓ Duration & severity of respiratory infections, improves oxygenation, adjunct in TB & COVID-19	Sex differences in vitamin C status & immunity unclear; more research needed	[18, 129–133]
**Vitamin D**	↑ Antimicrobial peptides (cathelicidin), modulates inflammation, Th1/Th2 balance	↓ Risk & severity of respiratory infections, beneficial in COVID-19	Females: ↑ VDR expression & higher levels (estrogen-mediated); males more deficient	[134–137]
**Zinc**	↑ Macrophage, neutrophil & T cell function, cytokine regulation, gut barrier & microbiota	↓ Infection severity, ↓ SARS-CoV-2 risk, reduced respiratory infections in elderly	No clear sex-specific data reported	[138–141]
**Selenium**	Regulates inflammation & antioxidant defenses; limits excessive oxidative stress	↓ Severity of viral infections (influenza, coxsackievirus)	No clear sex-specific data reported	[142]
**Omega-3** **LC-PUFAs (EPA/DHA)**	Modulates cytokines, reduces inflammation, enhances macrophage bacterial killing & resolution pathways	↓ Inflammation & improved clearance in TB models; ↓ risk of respiratory infections in elderly	No clear sex-specific data reported	[127, 143, 144]

#### Vitamin D

Vitamin D is a pleiotropic immunomodulator that contributes to host defense by regulating antimicrobial peptide production, maintaining epithelial integrity, and modulating inflammatory responses. Through vitamin D receptor signaling, it induces antimicrobial peptides such as cathelicidin and defensins, which enhance pathogen clearance while limiting excessive tissue-damaging inflammation [134]. The immunological effects of vitamin D are highly context dependent and may differ according to pathogen type and the immune mechanisms required for protection. In murine models of *L. amazonensis* infection, vitamin D deficiency unexpectedly improved lesion control by promoting Th1 responses and suppressing IL-10 production [135]. In this parasitic disease, where robust Th1 immunity is essential for parasite clearance, vitamin D–driven immunoregulation may dampen protective responses, highlighting that vitamin D supplementation may not be uniformly beneficial across all infectious contexts. These findings underscore the importance of pathogen- and immune-context–specific considerations, illustrating that the immunological effects of vitamin D supplementation depend on the type of pathogen and the immune mechanisms required for protection. Conversely, in respiratory infections and general immune health, vitamin D sufficiency is beneficial, enhancing antimicrobial peptide production (e.g. cathelicidin) and reducing infection risk [134]. A meta-analysis of over 11,000 participants found that regular vitamin D supplementation significantly reduced respiratory infection risk, particularly in individuals with low initial vitamin D levels [136]. Recent evidence also suggests that sex influences the immunological effects of vitamin D, particularly in the context of COVID‑19. Female generally exhibit higher circulating vitamin D levels and greater vitamin D receptor (VDR) expression than men, partly due to estrogen-mediated upregulation of VDR and vitamin D–activating enzymes [137]. In COVID‑19, these sex-based differences may contribute to the lower mortality observed in female, as adequate vitamin D status and estrogen signaling synergistically enhance antiviral defenses and temper inflammatory damage. Men, who often present with lower vitamin D levels and weaker estrogen-driven immune regulation, have been shown to be more susceptible to severe COVID‑19 outcomes, suggesting they may benefit from vitamin D supplementation [137].

#### Zinc

Zinc plays a central role in both innate and adaptive immunity. It supports the activity of immune cells such as macrophages, neutrophils, and T lymphocytes, and helps regulate cytokine responses [138]. Zinc deficiency has been associated with increased vulnerability to viral infections, including SARS-CoV-2, due to impaired immune signaling and inflammation control [139]. Supplementation, particularly when combined with vitamins C and D, improves mucosal integrity, supports gut microbiota, and reduces infection severity [140]. Furthermore, daily zinc gluconate supplementation (45 mg) in older adults significantly reduced the number of infections over a one-year period [141]. Selenium, although needed in trace amounts, regulates inflammation and antioxidant defense. Its deficiency worsens viral infection outcomes, as demonstrated in animal models with coxsackievirus and influenza [142]. Sex-related differences in zinc status have been reported, with males more frequently exhibiting zinc deficiency due to higher requirements, differences in dietary intake, and androgen-related effects on zinc metabolism [138, 145]. In viral infections, lower zinc levels in males may exacerbate impaired antiviral responses, potentially contributing to increased disease severity [146, 147]. However, clinical trials rarely stratify zinc supplementation outcomes by sex, limiting definitive conclusions [148]. Current evidence supports zinc supplementation as beneficial for immune defense in both sexes, particularly in zinc-deficient individuals, but highlights the need for sex-stratified analyses in future studies.

#### Omega- 3

Omega-3 long-chain polyunsaturated fatty acids (LC-PUFAs), particularly eicosapentaenoic acid (EPA) and docosahexaenoic acid (DHA) help regulate inflammation by incorporating into immune cell membranes, thereby influencing cytokine production serving as precursors for specialized pro-resolving mediators that actively terminate inflammation. These lipid mediators modulate cytokine production, macrophage polarization, and T-cell function, processes known to differ between males and females due to hormonal regulation of lipid metabolism and inflammatory signaling [149, 150]. In models of *M. tuberculosis* infection, EPA/DHA supplementation improved bacterial clearance and reduced inflammation [143]. In older adults, omega-3 supplementation has been linked to enhanced immune function and reduced risk of respiratory infections [144]. Although sex differences in lipid metabolism suggest that omega-3–derived immunomodulatory effects may differ between males and females, most infection-related supplementation studies do not stratify outcomes by sex.

### Feeding the microbiome: why sex matters?

Sex-based differences in the microbiome composition and function are pivotal in immunity and has undeniable implication in the development of chronic diseases [151]. Testosterone is commonly associated with reduced microbial diversity and may contribute to a more pro-inflammatory gut environment, promoting *Proteobacteria* and lowering the abundance of *Bacteroidota* [35, 152]. In contrast, estrogen supports a healthier gut microbiome by enhancing microbiota diversity and improving microbiota’s resilience following disturbances [58, 153]. It also fosters the growth of beneficial taxa such as *Lactobacillus*, *Bifidobacterium*, and *Akkermansia muciniphila*, and strengthens mucosal immunity by increasing IgA levels [154]. Progesterone, particularly during pregnancy, contributes to maternal and fetal health by reshaping the microbiota composition [155]. It promotes anti-inflammatory species such as *Faecalibacterium prausnitzii* and mitigates inflammation-induced microbial dysbiosis [156].

Exclusion diets are commonly used to support gut health. The low-FODMAP diet—low in fermentable oligosaccharides, disaccharides, monosaccharides, and polyols—is widely applied in inflammatory bowel conditions. By reducing the intake of fermentable carbohydrates, it helps alleviate symptoms such as bloating, gas, and diarrhea. These dietary interventions can also be personalized by identifying and eliminating individual symptom-triggering foods [157, 158]. Frequently excluded foods include dairy products, gluten, and highly processed foods [158]. Among anti-inflammatory dietary patterns, the Mediterranean diet stands out. Rich in fruits, vegetables, olive oil, and fish, and low in red meat, it has consistently been shown to reduce inflammation and improve metabolic and immune health [159, 160]. Importantly, studies report no significant sex-based differences in response to these dietary strategies, suggesting that biological sex does not primarily determine the efficacy of exclusion diets.

The use of probiotics—live microorganisms that promote gut health and systemic benefits—has gained increasing attention [158, 161]. Women often benefit from strains that support vaginal health, such as *Lactobacillus rhamnosus*, due to a higher susceptibility to urinary tract and yeast infections [162, 163]. In contrast, men may benefit from strains that support prostate health or address sex-specific health concerns, for instance in vitro studies suggest that the use of *Bifidobacterium longum* and *B. psychaerophilum* may mitigate benign prostate disease [164]. Nevertheless, foundational general like *Bifidobacterium* offer broad benefits across sexes. Estrogen plays a key role in promoting the growth and stability of beneficial *Lactobacillus* species, especially within the vaginal microbiome [165, 166]. Elevated estrogen levels during puberty and pregnancy favor the dominance of these protective bacteria. Progesterone also influences probiotic colonization and adherence, although its effects are subtler and more variable [167]. Hormonal declines, such as those occurring during menopause, can reduce *Lactobacillus* abundance, increasing susceptibility to infections and microbial imbalance [168]. At the intestinal level, probiotics are widely used to enhance gut health and support the integrity of the epithelial barrier. The most used genera in both men and women are *Lactobacillus* and *Bifidobacterium*, known to improve bowel transit, prevent and treat diarrhea, boost immune function, and aid in digestion and nutrient absorption [169]. Specific strains offer targeted benefits: *Lactobacillus acidophilus* helps prevent infections; *Bifidobacterium longum* and *Bifidobacterium animalis* support digestion, nutrient absorption, and toxin elimination; and *Saccharomyces boulardii* is effective in preventing antibiotic-associated and traveler’s diarrhea [170–173]. Despite sex-specific differences in how hormones affect the microbiome, the core probiotic strains used for intestinal health are effective in both sexes. However, colonization efficiency may differ, and these disparities can be addressed by optimizing probiotic formulations to account for sex-specific physiological factors.

Sex hormone–conditioned microbial configurations act as critical determinants of immune tolerance and pathogen resistance, thereby shaping susceptibility to autoimmunity and infection. Microbiomes enriched in commensal anaerobes such as *Faecalibacterium prausnitzii*, *Akkermansia muciniphila*, and *Bifidobacterium* enhance regulatory T-cell induction, maintain epithelial tight junctions, and increase IgA-mediated mucosal surveillance, mechanisms that protect against enteric pathogens while restraining autoimmune activation [174, 175]. In contrast, dysbiotic profiles characterized by reduced diversity and expansion of *Proteobacteria* increasing lipopolysaccharide burden, impair barrier integrity, and promote Th1/Th17 polarization, fostering chronic inflammatory states associated with autoimmune diseases such as IBD, RA, and MS [176–181]. These dysbiotic ecosystems also reduce colonization resistance, increasing susceptibility to infections including *Clostridium difficile* colitis, recurrent urinary tract infections, and respiratory viral illness, positioning the microbiome as a central mechanistic link between sex-specific microbial patterns and immune-mediated disease risk [182–185]. Collectively, these observations indicate that sex hormones shape microbiome configurations that, in turn, program immune tolerance and pathogen resistance, thereby influencing lifelong susceptibility to autoimmune and infectious diseases. Recognizing sex hormones as upstream modulators of microbiome–immune interactions is therefore critical for the development of precision-based strategies for disease prevention and therapeutic intervention.

## Clinical and therapeutic implications of sex differences in infection and inflammatory disease management

Recognizing sex-based differences in immune function is crucial for optimizing prevention and treatment strategies for infections and inflammatory diseases. Males, who generally exhibit weaker immune responses, may benefit from immune-enhancing therapies. In contrast, females, who typically display stronger immune activation, may require more precise anti-inflammatory treatments to avoid overactivation and reduce the risk of adverse effects or autoimmune complications. These divergent immunological profiles highlight the need for personalized, sex-specific strategies, including dietary patterns and nutritional supplementation and immunotherapies. Evidence from both clinical and experimental studies support this approach. A cohort study in Iran found that male patients with anthroponotic cutaneous leishmaniasis treated with meglumine antimoniate had a 1.54-fold higher risk of treatment failure compared to females, suggesting that sex-related differences in immune responses can affect therapeutic efficacy [186]. Despite such findings, most clinical trials still lack sex-disaggregated analysis, limiting the ability to tailor treatments based on biological differences. As mentioned above, estrogens tend to enhance, while androgens suppress key immune function [187]. This contributes to higher vaccine-induced antibody responses in females, often accompanied by increased reactogenicity, including fever, localized pain, and inflammation, which may affect compliance and vaccine confidence. To improve both efficacy and tolerability, sex-specific adaptation in vaccine dosing, intervals, or platforms (e.g., viral vector vs. inactivated virus) may be necessary to achieve optimal outcomes for all populations. Fathi et al.. advocate for sex-stratified clinical trials to better reflect these differences and ensure optimized outcomes across sexes [187]. The COVID-19 pandemic further highlighted these disparities. Males experienced more severe disease and higher mortality, while females, though better protected by vaccination, reported higher rates of side effects [187]. This underscores the importance of embedding sex-based analysis in all stages of therapeutic development, from preclinical studies to post-marketing surveillance. As an example of diet intersecting with vaccine outcomes, an Italian survey study, higher Mediterranean diet (MedDiet) adherence was associated with differences in SARS-CoV-2 vaccine side-effects, with women reporting distinct patterns (e.g., lymph-node enlargement) and severity profiles compared with men; overall findings supported a protective role of MedDiet and supporting sex-aware analysis of diet–vaccine interactions [188].

Studies involving gender-affirming hormone therapy (GAHT) have provided valuable insights into the immunomodulatory effects of sex hormones, independent of chromosomal sex. For example, testosterone therapy in transgender male is associated with reduced type I IFN responses and increased TNF-α signaling, resulting in a more pro-inflammatory immune profile [189, 190]. Conversely, estrogen therapy in transgender female promotes B cell activation and enhanced antibody production, consistent with increased vaccine responsiveness but potentially higher risk of autoimmune complications. The evidence highlights how hormonal status, beyond chromosomal makeup, shapes immune function and should inform vaccine strategies, pharmacotherapy, and personalized medicine. Importantly, the efficacy of immunomodulatory treatments, such as cytokine inhibitors or monoclonal antibodies, may also differ by sex, with some interventions showing greater efficacy in males [191]. Incorporating sex- and gender-informed insights into immune regulation is critical to achieving equitable and effective personalized therapies.

Sex- and hormone-driven differences also impact responses to therapy, particularly to immunomodulators and biologic agents frequently used in the management of immune-mediated diseases. Standard treatment typically starts with immunomodulators, such as methotrexate and glucocorticoids, followed by biologicals when necessary [99]. Dietary adjuncts can also modulate anti-inflammatory therapy. In rheumatoid arthritis, a meta-analysis found that omega-3 PUFA supplementation (> 2.7 g/day for > 3 months) reduced NSAID use, supporting diet-based adjuncts to conventional therapy; although trials were not sex-stratified, complementary evidence points to sex differences in omega-3 biology (e.g., EPA/DHA status and lipid mediator profiles), underscoring the need for sex-aware dosing and analysis in future RA trials [192].

Biologics target discrete inflammatory pathways to reduce disease activity and limit tissue damage, but they may increase infection risk and require careful monitoring [193]. As with disease expression, treatment effectiveness and adverse effects are modulated by sex and hormones. Treatment adherence to biologics is higher in males, often attributed to the greater frequency of adverse effects reported by females [194]. Pharmacokinetics are also influenced by hormonal milieu, affecting drug metabolism and clearance. In general, females need higher drug levels, increasing the risk of toxicity [195]. Males usually require higher doses due to faster drug clearance [195, 196]. Regarding side effects, females are more prone to systemic side effects (such as headaches, infusion reactions) while males present higher risk of serious infections and liver related adverse effects [197]. Finally, treatment adherence and disease perception differ between sexes: female tend to report more symptoms and greater quality-of-life impact, necessitating closer monitoring to assess the need for treatment adjustments, whereas [191, 198], male are more likely to underreport symptoms and show lower adherence to long-term therapies [198]. Incorporating sex and gender considerations into the design, monitoring, and personalization of immunotherapies is essential to optimize outcomes for all patients.

## Conclusion

Biological sex shapes how our immune system responds to infections, inflammation, and autoimmune diseases, influencing not only who gets sick but also how effectively treatments work. These differences arise from a complex interplay of genetics, hormones, and environmental factors, yet they are too often overlooked in research and clinical practice. The long‑standing underrepresentation of females in studies has led to treatment guidelines that assume a “one‑size‑fits‑all” approach, even though evidence clearly shows that immune responses and treatment outcomes can vary significantly between sexes. To truly advance precision medicine, sex stratification must be placed at the center of research on infectious and inflammatory diseases. Importantly, this shift is increasingly reinforced by evolving expectations from funding agencies and ethical review boards. Many national and international funding bodies now explicitly require researchers to address how sex and/or gender is considered in study design, analysis, and reporting, recognizing that failure to do so can limit scientific rigor, reproducibility, and translational relevance. Understanding how hormones, the microbiome, and other biological variables intersect with sex to shape immunity will enable us to design interventions that are both more effective and unbiased for everyone. Advances like AI‑driven recommendation systems offer exciting opportunities to translate these insights into personalized therapies and nutrition plans. But first, we must commit to research that recognizes and embraces sex differences, paving the way for more effective, equitable, and human‑centered care in immune‑related diseases.

## Data Availability

Not applicable.

## Abbreviations

ACPAs: Anti–citrullinated protein antibodies
AhR: Aryl hydrocarbon receptor
ATP: Adenosine triphosphate
BCAA: Branched–chain amino acids
CD: Cluster of differentiation
COVID: 19–Coronavirus disease 2019
CXCL: C–X–C motif chemokine ligand
DC: Dendritic cell
DHA: Docosahexaenoic acid
DHT: Dihydrotestosterone
EPA: Eicosapentaenoic acid
ERα: Estrogen receptor alpha
ETS1: E26 transformation–specific sequence–1
FODMAP: Fermentable oligosaccharides, disaccharides, monosaccharides, and polyols
FOXP3: Forkhead box protein P3
GAHT: Gender–affirming hormone therapy
IBD: Inflammatory bowel disease
IFN: Interferon
IL: Interleukin
ILC: Innate lymphoid cell
iNOS: Inducible nitric oxide synthase
LC: PUFA–Long–chain polyunsaturated fatty acid
MHC: Major histocompatibility complex
MS: Multiple sclerosis
NF: κB–Nuclear factor kappa–light–chain–enhancer of activated B cells
NK: Natural killer
PBMC: Peripheral blood mononuclear cell
pDC: Plasmacytoid dendritic cell
RA: Rheumatoid arthritis
RF: Rheumatoid factor
ROS: Reactive oxygen species
SARS: CoV–2–Severe acute respiratory syndrome coronavirus 2
SLE: Systemic lupus erythematosus
TB: Tuberculosis
TGF: β–Transforming growth factor beta
Th: T helper
TLR: Toll–like receptor
TNF: α–Tumor necrosis factor alpha
Treg: Regulatory T cell
UC: Ulcerative colitis
VDR: Vitamin D receptor
VL: Visceral leishmaniasis
XIST: X–inactive specific transcript
